# Comparisons of early vascular reactions in biodegradable and durable polymer-based drug-eluting stents in the porcine coronary artery

**DOI:** 10.1371/journal.pone.0209841

**Published:** 2019-01-10

**Authors:** Takeshi Ijichi, Gaku Nakazawa, Sho Torii, Hirofumi Nagamatsu, Ayako Yoshikawa, Junko Souba, Atsushi Isobe, Hitomi Hagiwara, Yuji Ikari

**Affiliations:** 1 Department of Cardiology, Tokai University School of Medicine, Kanagawa, Japan; 2 TERUMO Corporation Evaluation Center, Kanagawa, Japan; Nagoya University, JAPAN

## Abstract

Current drug-eluting stents have abluminal polymer coating; however, thrombus formation in these compared with that in uniformly coated stents remains controversial. We evaluated thrombus formation and early endothelialization after using abluminal biodegradable polymer-coated sirolimus- (BP-SES), and everolimus-eluting stents (BP-EES) versus a durable polymer-coated everolimus-eluting stent (DP-EES) in an *in vivo* setting. BP-SES, BP-EES, and DP-EES (n = 6 each) were implanted in coronary arteries of 12 mini-pigs that were then sacrificed after 7 and 10 days. Stents were stained with hematoxylin and eosin, and a combined Verhoeff and Masson trichrome stain. Areas of fibrin deposition were digitally detected and measured with off-line morphometric software. Stents were investigated for re-endothelialization by transmission electron microscopy. At 7 days, histological analysis revealed the lowest area of fibrin deposition in BP-SES (BP-SES vs. BP-EES vs. DP-EES; 0.10 ± 0.06 mm^2^ vs. 0.15 ± 0.07 mm^2^ vs. 0.19 ± 0.06 mm^2^, *p* = 0.0004). At 10 days, the area of fibrin deposition was significantly greater in DP-EES (0.13 ± 0.04 mm^2^ vs. 0.14 ± 0.05 mm^2^ vs. 0.19 ± 0.08 mm^2^, *p* = 0.007). Endothelial cells in BP-SES demonstrated a significantly greater number of tight junctions than those in DP-EES according to by transmission electron microscopy for both days (*p*<0.05). Various parameters, including an inflammatory reaction and neointimal formation, were comparable among the groups at 7 and 10 days. An abluminal biodegradable polymer-coated SES showed the least fibrin deposition and greatest endothelial cell recovery at an early stage following implantation in the coronary arteries of mini-pigs.

## Introduction

Stent thrombosis (ST) is known as a fatal complication after percutaneous coronary intervention [1, 2]. Although delayed endothelial healing was considered to be the main cause of a late ST for the first generation of drug-eluting stents (DES), recent studies hypothesized that an abnormal vascular reaction, such as neoatherosclerosis and severe inflammation, contributed to a very late ST [3, 4]. However, despite the dramatic improvement of arterial healing for newer DES [5], early ST is still observed in a clinical setting. Therefore, it is important to understand the early vascular reaction that leads to ST. In patients presenting with early ST, Nakano et al. demonstrated that necrotic core prolapse, medial tear and incomplete strut apposition were associated with the occurrence of ST [6].

Meanwhile, a particular durable fluoropolymer-coated everolimus-eluting stent (DP-EES) showed clinically low rates of ST compared to other similar stents [7]. Previous research indicated this fluoropolymer coating may have an anti-thrombotic effect even when compared with a bare metal surface [8]. However, most of the newest generation of drug-eluting stents have an abluminal polymer coating applied, meaning the luminal surface is bare metal [9, 10]; therefore, the anti-thrombotic effect of stents with an abluminal polymer coating may be weaker compared to that of stents with a uniform fluoropolymer coating [11]. However, *in vivo* studies have not been carried out to confirm the results of *in vitro* or *ex vivo* studies.

The aim of this study was to evaluate thrombus formation and early endothelialization following the implantation of abluminal biodegradable polymer-coated sirolimus (BP-SES) and everolimus-eluting (BP-EES) stents versus a uniform durable fluoropolymer-coated DP-EES in a porcine coronary artery model.

## Materials and methods

### Study design

Fourteen mini-pigs (Science Breeding Co., Ltd., Chiba, Japan) were used in this study. Three different types of drug eluting stent (DES) were implanted: DP-EES (XIENCE Xpedition; Abbott Vascular, Tokyo, Japan), BP-SES (Ultimaster; Terumo Medical Corp., Tokyo, Japan), and BP-EES (Synergy; Boston Scientific Japan, Tokyo, Japan), with one of each type implanted into each pig (one stent per vessel). The DP-EES is an everolimus-eluting stent with a uniform coating of durable fluoropolymer. The BP-SES has an abluminal coating with a matrix containing sirolimus and poly DL-lactide-co-caprolactone [12], whereas the BP-EES has an abluminal coating of a matrix containing everolimus and poly DL-lactide-co-caprolactone [13]. Prior research indicated that re-endothelialization was observed from 7 days to 10 days after these DES implantations by an optical microscope [14], likewise, endothelial cells could be detected on the stent struts 10 days following the implantations by transmission electron microscope (TEM). Therefore, animals were sacrificed at 7 and 10 days for investigating early vascular reaction. The number of the animals was derived from a previous study [15]. Following euthanasia, stents were subjected to histologic examination to analyze thrombus formation and vascular responses. This study was approved and performed according to the guidelines of the Institutional Animal Care and Use Committee of R&D Headquarters at Terumo Corporation.

### Animal preparation and procedures

The current experiment was based on Food and Drug Administration guidance. Briefly, the clinical condition of the animals was observed at least once daily, except for non-work days, during the period from the day before the implantation day to the scheduled necropsy day. The animals were visually observed and observations including external appearance, appetite, respiratory condition, nutrition status, and fecal condition were recorded, as well as any other abnormalities. All animals were individually housed in cages for pigs. Approximately 1.6 kg of MP-A (Oriental Yeast Co., Ltd.) was provided once daily.

All animals were administered oral clopidogrel (75 mg/day) and aspirin (330 mg/day) three days before the procedure, and these medications were continued until the day before euthanasia. The animals were also fasted for more than 15 hours from the day before the procedures. After anesthesia with 2.5% sevoflurane, surgical access was obtained via a carotid artery using general sterile techniques. During cardiac catheterization, heparin (300 IU/kg) was given to maintain an activated clotting time of 250–300 seconds. Vessel allocation to experimental groups was predetermined to distribute the different stent types equally in three different coronary arteries, with a targeted oversize of 1.1–1.2:1. After stent implantation, coronary angiography was performed to check vessel patency and the absence of coronary arterial dissection.

The animals were euthanized under general anesthesia at 7 and 10 days. Hearts were excised and pressure perfused with 0.9% saline until cleared of blood, followed by pressure perfusion fixation in 2% paraformaldehyde and 1.25% glutaraldehyde until hardening of the heart muscle was clearly perceptible.

### Histologic preparation and assessments

The stented arteries were fixed in 1% osmium, and embedded in Quetol-651 resin. After polymerization, sections were divided into proximal, middle and distal blocks [16] sectioned serially, and stained with hematoxylin and eosin and a combined Verhoeff and Masson trichrome stain. The cross-sectional areas of internal elastic lamina (IEL) were measured using digital morphometry (Olympus Cellsens platform, Tokyo, Japan) for each stented section.

**Fig 1:**
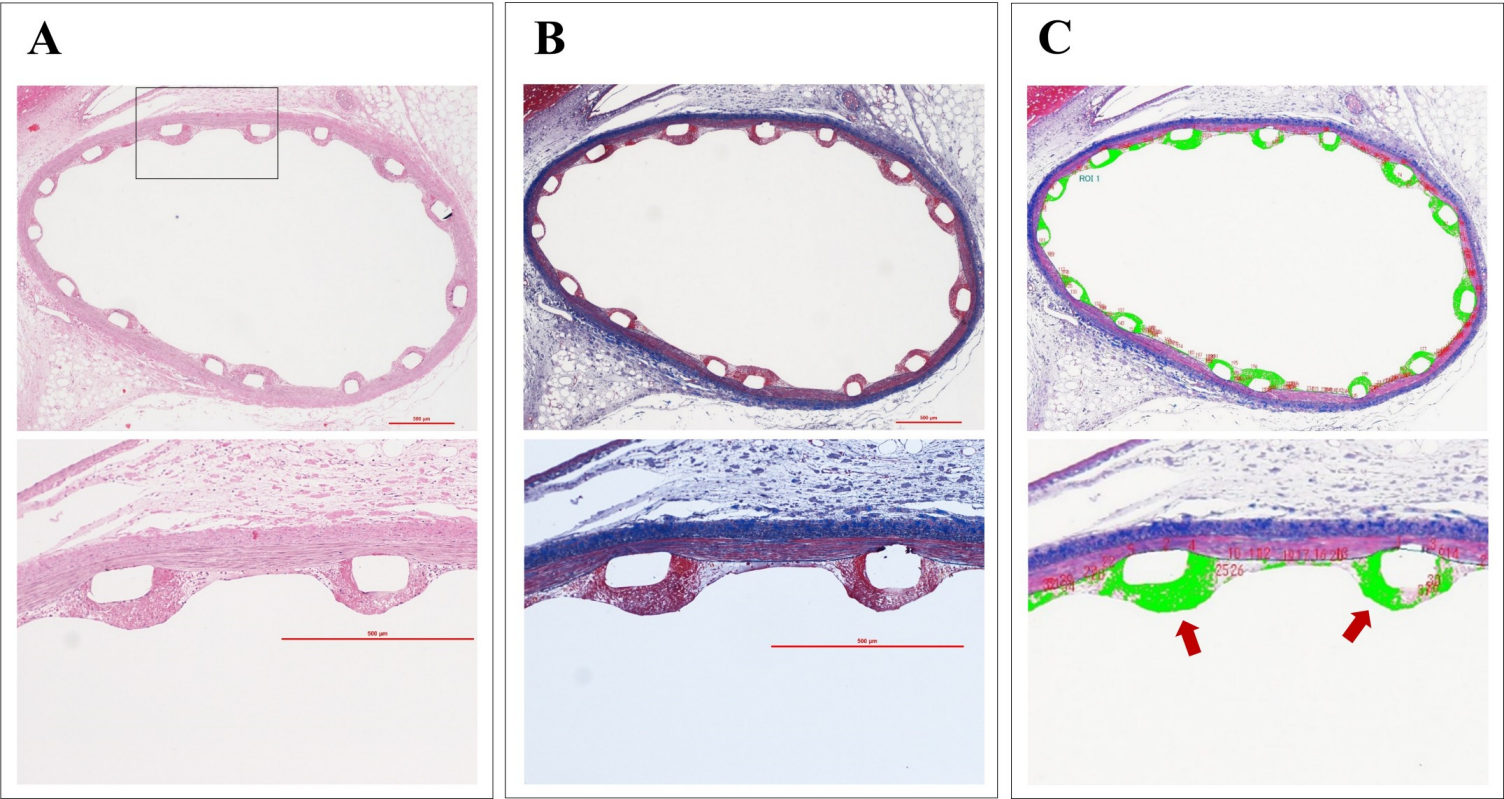
Quantification method of fibrin deposition surrounding stent struts. (A–B) Representative histologic sections stained with hematoxylin and eosin, and a combined Verhoeff and Masson trichrome stains, showing fibrin deposition surrounding stent struts. Fibrin was identified as an intense, homogenous red stain in a combined Verhoeff and Masson trichrome stains (B-lower panel). (C) Areas of neointimal fibrin deposition were digitally detected as green areas (red arrows) and measured with off-line morphometric software (WinROOF image-processing software, Version 6).

Histomorphometric analysis was performed to quantify fibrin deposition and to assess arterial inflammation. An overview of quantification method of fibrin deposition was shown in Fig 1. Briefly, fibrin was identified as an intense, homogenous red stain and we quantified the areas with a combined Verhoeff and Masson trichrome stain. Areas of fibrin deposition in sections with the combined Verhoeff and Masson trichrome stain were digitally detected and measured with off-line morphometric software (WinROOF image-processing software, Version 6; Mitani Corp., Tokyo, Japan). For each stent section, ordinal data were collected from histomorphometric findings around the stent struts. These were expressed as a percentage of the total number of struts in each section. An overall neointimal inflammation value was scored for each section, as previously described [17].

### Preparation and assessment of transmission electron microscope

The analysis of TEM images was performed by an independent assessor (Food and Drug Safety Center-Hatano Research Institute). All struts were selected from hematoxylin and eosin-stained specimens for the detailed analysis of endothelial cells by TEM. The present study used TEM to observe each strut located on the myocardium side. Moreover, struts were selected from locations with a moderate degree of strut coverage. Serial sections were re-embedded in Quetol-812 in a supinate position. The struts were prepared as ultrathin sections, and we performed double staining using uranyl acetate and lead citrate.

### Statistics

JMP for Windows version 9.0.2 (SAS Institute Inc., Cary, NC, USA) and EXSUS version 7.7.1 (CAC EXICARE Corporation, Tokyo, Japan) were used for the analysis of histopathological findings. Data were expressed as mean ± standard deviation for continuous variables, and as percentages for categorical variables. Continuous variables were checked for a normal distribution using Bartlett’s test for equal variances. Statistical comparisons were performed using an ANOVA test with Dunnett’s post hoc correction when data sets were normally distributed or Kruskal–Wallis tests with a Steel test in the event of the non-parametric distribution of data. Nonparametric score data, including the area of fibrin and neointimal inflammation were compared using a Mann–Whitney *U* test or Fisher’s exact test. Findings graded by a TEM observation at 7 and 10 days were compared using a Mann–Whitney *U* test, and the total value of positive grades were analyzed by Fisher’s exact test. A *p* value <0.05 was considered statistically significant.

## Results

### Stent implantation

Stent implantation was successfully performed into three major coronary arteries of each of 14 pigs without any differences in quantitative coronary analyses. Two animals died unexpectedly leaving a total of 36 implanted stents (in 12 pigs) available for follow-up at 7 and 10 days–divided into two groups (6 pigs for each time point). One animal was dead at 3 days after implantation due to anesthesia during surgical treatment of the skin wound due to removing the suture. After necropsy, no abnormal sites of the heart and other organs were found. Another animal was found dead at 7 days before euthanization because of the faded color at the apical area of the heart thought to be myocardial infarction that was observed at the necropsy.

### Histomorphometric assessment and measurements

**Fig 2:**
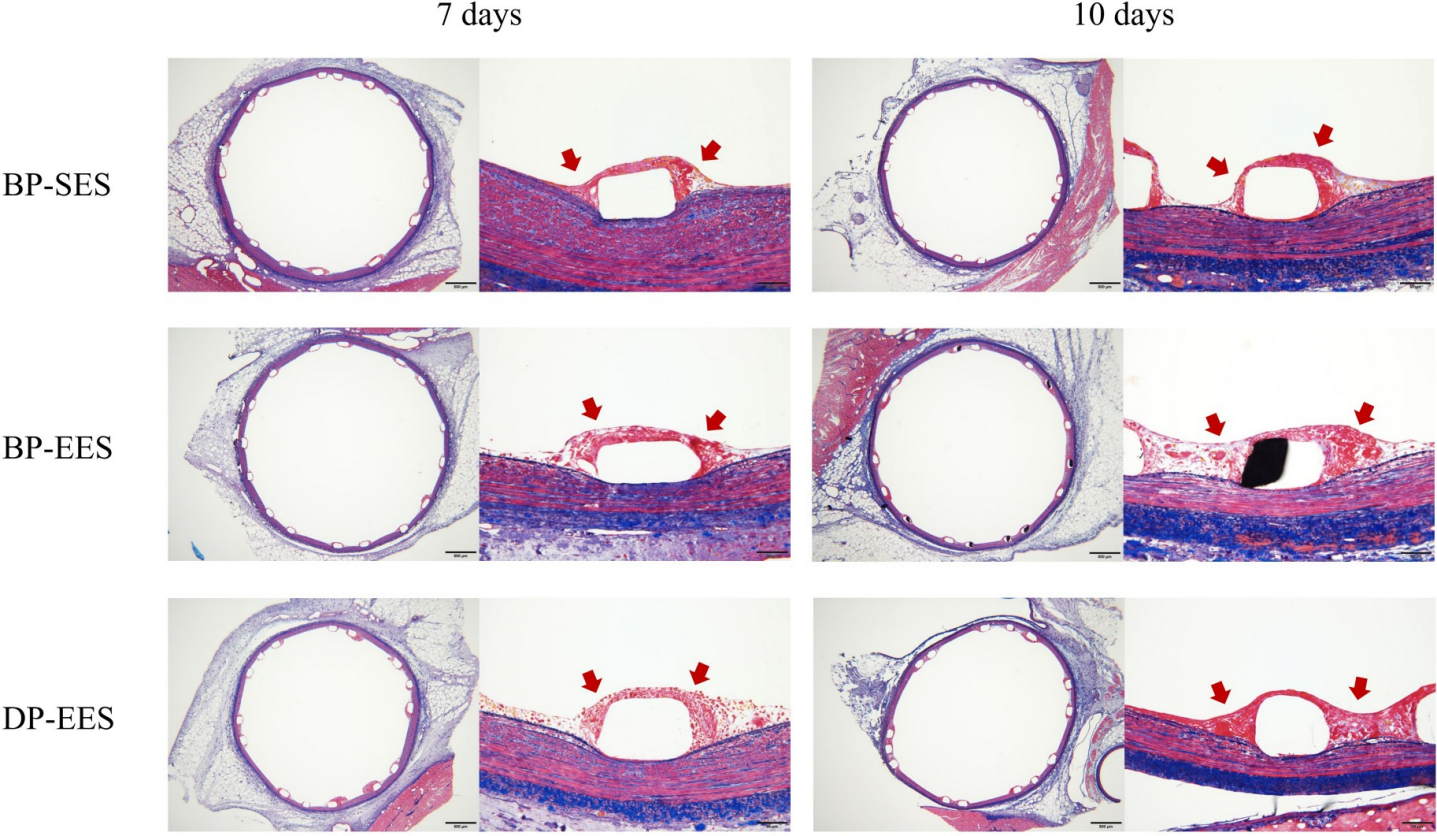
Representative histologic sections stained with a combined Verhoeff and Masson trichrome stains at 7 and 10 days. Sections show low power (4x) and high power (20x) magnification following a combined Verhoeff and Masson trichrome staining. These images showed fibrin deposition (red arrows) around stent struts.

**Fig 3:**
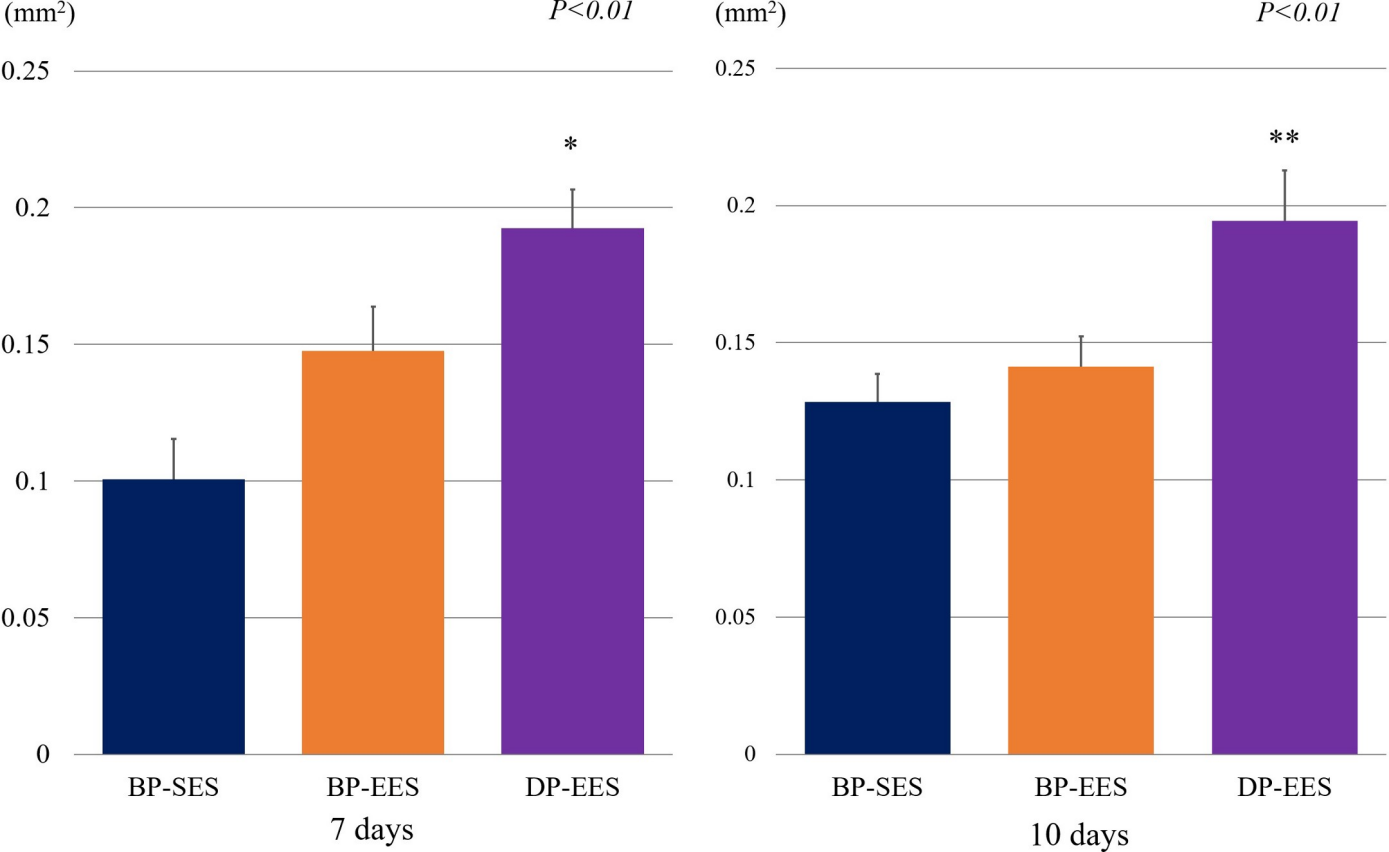
Comparison of areas of fibrin deposition across stent types. Areas of fibrin deposition were significantly greater for DP-EES when compared to BP-SES for both days. A significant difference in areas of fibrin deposition between BP-SES and BP-EES were not found. **p<0.01 versus BP-SES group.

**Fig 4:**
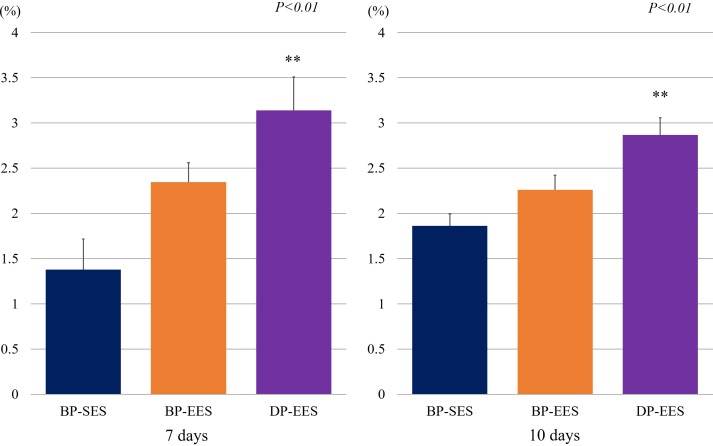
Comparison of percentage fibrin to internal elastic lamina areas across stent types. Similar to the results of Fig 3, significant differences between BP-SES and DP-EES with regard to the ratio of fibrin area to IEL areas for both days were noted. Although the ratio for BP-EES was numerically greater than that of BP-SES, a significant difference was not noted. **p<0.01 versus BP-SES group.

The representative histologic images and results of histomorphometric analyses are shown in Fig 2 and Table 1. Areas of IEL were similar across the study groups at each timepoint. There were no significant histopathological differences between BP-SES and BP-EES. The DP-EES group revealed a significantly greater fibrin area compared with BP-SES at each timepoint (Table 1 and Fig 3; *p*<0.01). Similar to fibrin area results, significant differences between BP-SES and DP-EES in the ratio of fibrin to IEL areas were noted (Table 1 and Fig 4; *p*<0.01). The inflammation score was largest for DP-EES, followed by BP-EES and BP-SES (Table 1; 1.14 ± 0.22%, 1.06 ± 0.18%, and 0.93 ± 0.23%, respectively; *p*<0.05) at 7 days, but did not differ significantly among the groups at 10 days.

**Table 1:** Results of Histomorphometric analysis at 7 and 10 day.

	7 days				10 days			
	BP-SES	BP-EES	DP-EES	p Value	BP-SES	BP-EES	DP-EES	p Value
IEL area (mm^2^)	7.40±0.53	6.94±1.87	6.78±2.01	N.S.	6.91±1.17	6.28±0.86	6.50±1.51	N.S.
Fibrin area (mm^2^)	0.10±0.06	0.15±0.07	0.19±0.06**	<0.01	0.13±0.04	0.14±0.05	0.19±0.08**	<0.05
Fibrin area / IEL area (%)	1.4±0.9	2.3±1.4	3.1±1.6**	<0.01	1.9±0.6	2.3±0.7	2.9±0.8**	<0.01
Inflammation score	0.93±0.23	1.06±0.18	1.14±0.22*	<0.05	1.01±0.15	0.96±0.20	1.07±0.37	N.S.

Each group n = 6

Mean ± standard deviation,

*p<0.05,

**p<0.01 versus BP-SES group

IEL = Internal elastic lamina; BP-SES = biodegradable polymer–coated sirolimus-eluting stent; BP-EES = biodegradable polymer–coated everolimus-eluting stent; DP-EES = durable polymer–coated everolimus-eluting stent; N.S. = not significant

### Assessment of tissue characteristics by transmission electron microscopy

**Fig 5:**
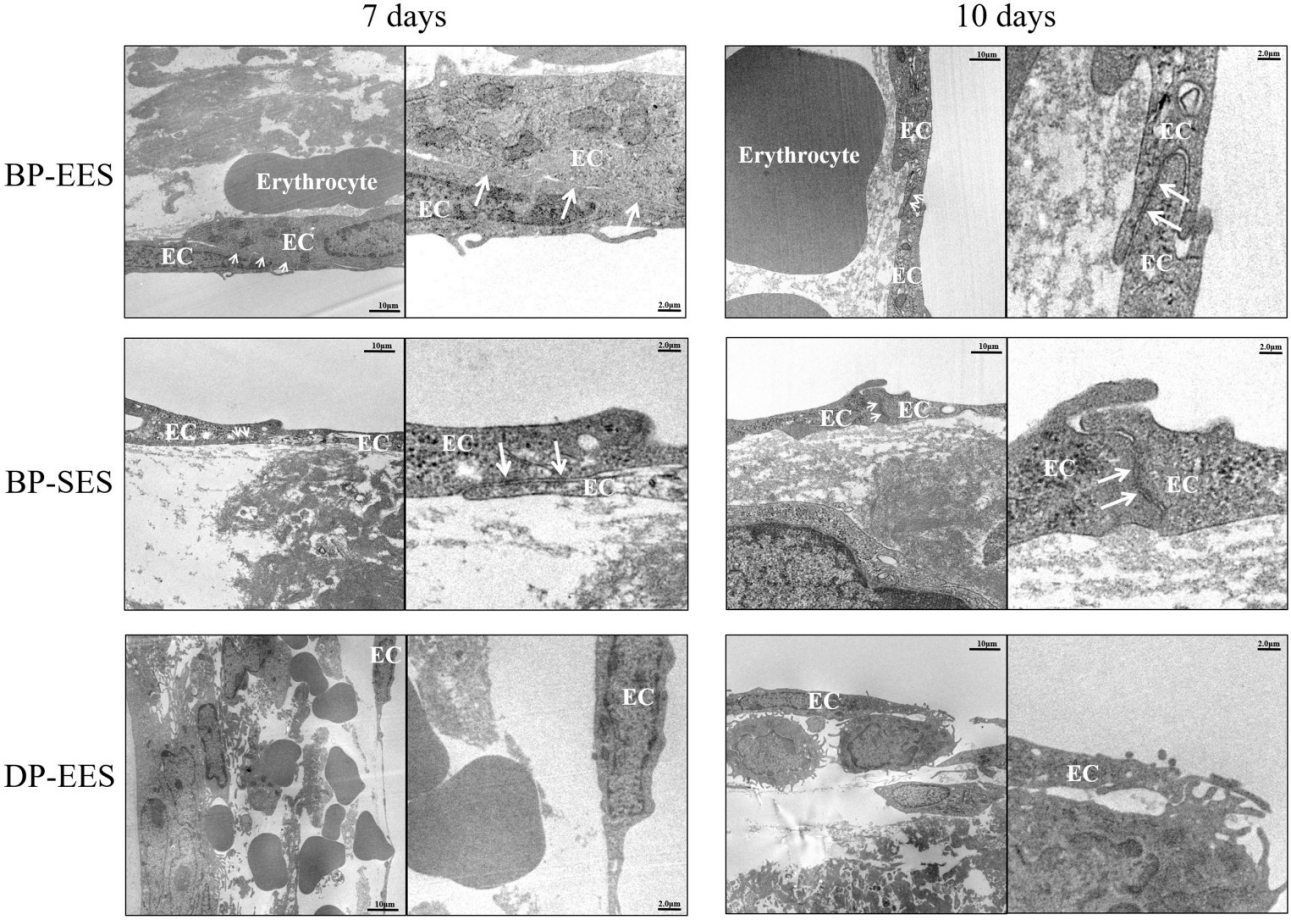
Representative images of transmission electron microscope at 7 and 10 days. Stents were also investigated for re-endothelialization by transmission electron microscope (TEM) at the Food and Drug Safety Center–Hatano Research Institute. All struts were selected from hematoxylin and eosin-stained specimens for detailed analysis by TEM. TEM images showed endothelial cells (EC) and tight junctions (white arrows) around stent struts.

Fig 5 shows representative TEM images at 7 and 10 days. Results of characteristic findings in tissues by TEM at 7 days are shown in Table 2. While qualitative TEM analysis demonstrated a significantly decrease frequency of erythrocytes around the stent struts for BP-EES compared to BP-SES (Table 2; *p*<0.01, *p*<0.05), significant differences in other findings around the stent struts were not noted with TEM. DP-EES demonstrated significantly decreased numbers of tight junctions in endothelial cells compared with BP-SES at each timepoint (Tables 2 and 3; *p*<0.05 for both).

**Table 2:** Transmission Electron Microscopic Findings at 7 days.

	Group	BP-SES						BP-EES						DP-EES					
Findings	Grade	-	±	+	2+	3+	P	-	±	+	2+	3+	P	-	±	+	2+	3+	P
**Around stent strut**	Inflammatory cell	16	1	1	0	0		18	0	0	0	0		11	6	1	0	0	
	Erythrocyte	3	8	7	0	0		10^*^	7	1	0	0		7	6	5	0	0	
**Endothelial cell**	Inflammatory cell	17	1	0	0	0		17	1	0	0	0		15	3	0	0	0	
	Tight junction	0					18	2					16	5^#^					13

Grade)—: No abnormal changes ±: Very slight +: Slight 2+: Moderate 3+: Marked P: Non-graded change

Numerals represent the number of coronary arteries.

*p<0.01: Significantly different from BP-SES group (Mann-Whitney U test).

#p<0.05: Significantly different from BP-SES group (Fischer exact test).

BP-SES = biodegradable polymer–coated sirolimus-eluting stent; BP-EES = biodegradable polymer–coated everolimus-eluting stent; DP-EES = durable polymer–coated everolimus-eluting stent

**Table 3:** Transmission Electron Microscopic Findings at 10 days.

	Group	BP-SES						BP-EES						DP-EES					
Findings	Grade	-	±	+	2+	3+	P	-	±	+	2+	3+	P	-	±	+	2+	3+	P
**Around stent strut**	Inflammatory cell	16	2	0	0	0		15	3	0	0	0		16	2	0	0	0	
	Erythrocyte	8	7	2	1	0		8	8	2	0	0		10	7	1	0	0	
**Endothelial cell**	Inflammatory cell	17	1	0	0	0		15	3	0	0	0		18	0	0	0	0	
	Tight junction	1					17	4					14	6^#^					12

Grade)—: No abnormal changes ±: Very slight +: Slight 2+: Moderate 3+: Marked P: Non-graded change

Numerals represent the number of coronary arteries.

#*p*<0.05: Significantly different from BP-SES group (Fischer exact test)

BP-SES = biodegradable polymer–coated sirolimus-eluting stent; BP-EES = biodegradable polymer–coated everolimus-eluting stent; DP-EES = durable polymer–coated everolimus-eluting stent

## Discussion

The major findings of the current animal study are: 1) decreased fibrin deposition at 7 and 10 days following stent implantation for BP-SES compared to DP-EES; and 2) a consistent decrease in tight junctions of endothelial cells with DP-EES, whereas BP-SES and BP-EES showed good formation of tight junctions, even at an early stage.

Fibrin deposition around stent struts is considered to occur by the initial aggregation of fibrin at sites of stent implantation due to vessel injury, inflammation and changed blood flow [11, 14, 18]. Recent *in vitro* studies demonstrated that the fluoropolymer of DP-EES had a high anti-thrombogenic effect [11, 19]. In a study by Otsuka et al., DP-EES showed less platelet aggregation than other DES with biodegradable polymer coatings such as BioMatrix (Biosensors, Newport Beach, CA), Synergy (Boston Scientific, Natick, MA), Nobori (Terumo, Tokyo, Japan), and Orsiro (Biotronik AG, Bülach, Switzerland) in an *ex vivo* swine arteriovenous shunt model [19]. According to the EXAMINATION trial, the prevalence of definite stent thrombosis in DP-EES was significantly lower than in bare metal stents (at 30 days, 0.4% vs. 1.6%, *p* = 0.0204; at 12 months, 0.5% vs. 1.9%, *p* = 0.0183) [20], which supports the hypothesis of an anti-thrombotic effect of fluorinated polymer. Moreover, definite stent thrombosis was decreased in DP-EES compared with other first and second generation drug-eluting stents [7]. By contrast, BP-SES had less amount of fibrin deposition rather than DP-EES in the present study, despite BP-SES applied abluminal polymer coating (a bare metal stent surface). Kolandaivelu et al. reported early clotting was reduced by DP-EES compared with a bare metal stent in ex vivo flow loops (0.76 ± 0.02 vs. 1.00 ± 0.15, *p*<0.002) and strut thickness was also associated with thrombus formation by stent-induced flow disruption in *ex vivo* flow loops with thin struts (81 μm) vs. thick struts (162 μm) [11]. Conversely, other clinical trial has shown biodegradable polymer biolimus-eluting BioMatrix stents (120 μm) had similar safety outcome regarding to stent thrombosis compared with DP-EES (81 μm) in the total and propensity score-matched populations (0.4% versus 0.4%, *p* = 0.99) [21]. In the present study, stent struts were almost similar in all groups (BP-SES, 80 μm; BP-EES, 74 μm), which speculate that another factor may influence the platelet aggregation with an *in vivo* setting.

Endothelial cells produce prostaglandin I2 to inhibit platelet aggregation and the blood coagulation reaction, and to activate the fibrinolytic system to prevent thrombus formation [22], whereas injury and peeling of endothelial cells by physical stent stress causes platelet aggregation and blood coagulation at a stent site [23]. The present study showed that BP-SES had less fibrin deposition compared with DP-EES *in vivo*, which may indicate the fluoropolymer-coated stent is not necessarily more anti-thrombogenic than the newest stents with an abluminal coating in an *in vivo* setting. This indication might be supported by less inflammatory reaction, which induced platelet aggregation, in BP-SES than in DP-EES at 7 days (Tables 1 and 2) [8, 17]. Consistent with the present findings, a recent clinical trial did not demonstrate a significant difference in stent thrombosis between BP-SES and DP-EES (acute ST, 0.0% vs. 0.0%, *p* = 1.0; subacute ST, 0.5% vs. 0.4%, *p* = 0.65) [24]. It has been reported that the inflammatory cells remaining around stent struts decreased with time [14]. For this reason, the inflammation score did not differ significantly among the groups at 10 days.

Many kinds of differences exist among DES such as drug, polymer-coating method and platform designs; therefore various factors may influence vascular healing at stent sites. Immunosuppressive agents and anti-cancer agents are used as drugs in DES to inhibit smooth muscle proliferation. Compared to circumferential coating, the concept of abluminal coating is expected to reduce the drug load used [12, 13]. With respect to the pharmacokinetic properties of DES, a median maximal concentration of drug from BP-SES is lower than for DP-EES in peripheral blood samples [9]. The pharmacokinetics of sirolimus with a biodegradable abluminal coating may result in the alleviation of vascular inflammation and faster endothelial maturation.

Furthermore, it is presumed that re-endothelialization may be influenced by the existence of drug on the luminal side; therefore the difference in coating method, such as abluminal coating, was probably related to the greater extent of re-endothelialization in an *in vivo* setting with the current animal model [12, 13, 25, 26]. In addition, a preclinical study comparing different “limus” drugs using the same stent platform and polymer showed the greatest fibrin deposition in everolimus-eluting stents compared with sirolimus- and zotarolimus-eluting stents [27]. In order to assess the maturation of endothelial cells, the present study used TEM which was used to image the cytoplasm and formation of tight junctions around endothelial cells. Otsuka et al. described how tight junctions are a part of cell-to-cell junctions that maintain the integrity of endothelium and regulate endothelial permeability and fragility, as well as endothelial cell growth and survival [28]. Poorly formed cell-to-cell junctions are related to the incompetency of the regenerated endothelium in stented regions [28, 29]. In the present TEM observation at 7 days, the DP-EES group showed a lower degree and frequency of formation of tight junctions compared to the BP-SES group, suggesting that the recovery of the endothelial layer at a very early time point was delayed in DP-EES. As a result, it can be assumed that the faster speed of maturation of endothelial cells contributed to the decrease in fibrin deposition seen in the BP-SES group.

There were several limitations in the present study. First, the favorable vascular compatibility of a biodegradable polymer in comparison to a permanent polymer in healthy porcine coronary arteries cannot be extrapolated to diseased human coronary arteries, where disease conditions and atherosclerotic plaque composition may influence polymer degradation and inflammatory responses [14]. Second, the present study examined particular coating methodology and load of poly DL-lactide-co-caprolactone and durable fluoropolymer coatings, respectively, and the results may not be generalizable to other polymer coating methodology and load doses. Third, the study was carried out with a limited number of samples, and therefore the lack of significance may be related to the limited numbers in this study and should be addressed in future histopathological studies with sequential follow-up.

## Conclusions

Our study demonstrated that an abluminal polymer-coated BP-SES with a luminal surface of bare metal showed less fibrin deposition and greater endothelial cell recovery compared with the uniform fluoropolymer coating of DP-EES, suggesting that BP-SES may potentially prevent early stent thrombosis.
